# Selecting an appropriate strategy to make quality 7.1 % chlorhexidine digluconate accessible for umbilical cord care

**DOI:** 10.1186/s40545-016-0063-9

**Published:** 2016-04-08

**Authors:** Mutsumi Metzler, Patricia S. Coffey

**Affiliations:** PATH, 2201 Westlake Avenue, Suite 200, Seattle, WA 98121 USA

**Keywords:** Essential medicines, Local manufacturing, Product availability, Quality assurance, Chlorhexidine, Neonatal mortality, Cord care

## Abstract

Achieving increased access to medicines in low- and middle-income countries is a complex issue that requires a holistic approach. Choosing an appropriate manufacturing strategy that can ensure a sustainable supply of these medicines is an essential component of that approach. The Chlorhexidine Working Group, a consortium of more than 25 international organizations, donors, and manufacturers led by PATH, has been working to increase access to 7.1 % chlorhexidine digluconate for umbilical cord care in low- and middle-income countries to reduce neonatal mortality due to infection. The working group initially considered two strategies for manufacture of this commodity: (1) production and global distribution by a multinational company; and (2) production and regional distribution by locally owned companies or subsidiaries of multinational companies based in low- and middle-income countries. Local production may be beneficial to public health and economic development in these countries, yet capability and capacity of pharmaceutical manufacturers, regulatory and legal provisions, and market factors must be carefully assessed and addressed to ensure that local production is the correct strategy and that it contributes to improved access to the medicine. To date, this effort to implement a local production strategy has resulted in successful registration of 7.1 % chlorhexidine digluconate for umbilical cord care by manufacturers in Bangladesh, Kenya, Nepal, and Nigeria. Additionally, the product is now available in domestic and export markets.

## Background

In 1975, the World Health Assembly asked the World Health Organization (WHO) to assist member states to select and procure essential drugs, assuring good quality and reasonable cost. Two years later, the first WHO model list of essential drugs was established. Since then WHO has been addressing the need to increase equitable access to and rational use of quality assured essential medicines through avenues such as the WHO Revised Drug Strategy and a prequalification program [1]. However, in many low- and middle-income countries (LMICs), the availability of generic medicines in the public sector is still limited; for example, availability in the African region is only 40 % [2]. Further, even the least expensive generic medicines available in the African public sector are often priced higher than the international reference prices for the same generic medicines [2].

Severe infection is one of the top three causes of newborn deaths worldwide, claiming approximately 15 % of all neonatal deaths each year [3]. A baby’s newly cut umbilical cord can be an entry point for bacteria, leading to cord infection and potentially life-threatening sepsis. 7.1 % chlorhexidine digluconate (delivering 4 % chlorhexidine) is a generic antiseptic that is topically applied to the newly cut umbilical cord in order to prevent neonatal infection. Published data support the use of 7.1 % chlorhexidine digluconate for cord cleansing as a feasible, acceptable, efficacious, safe, and cost-effective intervention to reduce neonatal mortality in settings where poor hygiene and high neonatal mortality are issues [4, 5].

In 2013, 7.1 % chlorhexidine digluconate was included in the World Health Organization (WHO) Model List of Essential Medicines for Children under Specific Medicines for Neonatal Care. In 2014, WHO recommended daily application of 7.1 % chlorhexidine digluconate for the first week of life to babies born at home in settings with high neonatal mortality. The Chlorhexidine Working Group (CWG) [6], a consortium of more than 25 international organizations led by PATH, has been working to increase access to this evidence-based intervention in LMICs.

Currently the WHO prequalification program evaluates medicinal products used for HIV/AIDS, malaria, tuberculosis, neglected tropical disease, and reproductive health, as well as diagnostics, vaccines and immunization devices. The program also provides technical assistance to build quality assurance and manufacturing capacity at the local country level to ensure that quality product is available for purchase. This type of prequalification system, however, has several limitations. The process is resource intensive and also puts a high burden on small the manufacturer in terms of application costs, which could serve to restrict product availability and cause global shortage. Instead a harmonized, risk-based approach could be used to maximize quality assurance. In 2011, the joint stakeholder meeting organized by WHO and Global Fund categorized essential medicines into high, medium, and low risks based on therapeutic importance, pharmacologic characteristics, complexity of manufacturing procedures, and requirements associated with their formulation or dosage form and proposed to support quality assurance in procurement with increased rigor of evaluations for low-risk medicines rather than WHO prequalification [7].

This risk-based approach denotes 7.1 % chlorhexidine digluconate, which is topical antiseptic, as a low-risk generic drug and therefore its quality could be assured with support for procurers and in-country regulatory bodies. However, a key consideration should be given to how to effectively and efficiently achieve this. Because its market value and production volume are small, manufacturers may not be incentivized to produce 7.1 % chlorhexidine digluconate. The lack of a centralized procurement mechanism or pooled procurement system for essential newborn drugs also does not lend itself to motivating manufacturers to invest in production of this type of medicine.

Therefore, determination of an appropriate manufacturing strategy is key to ensuring availability of high-quality product.

### Strategic selection of a potential manufacturing strategy

Achieving increased access to medicines in LMICs is a complex issue that requires a holistic approach. Choosing an appropriate manufacturing strategy that can ensure a sustainable supply of these medicines is an essential component of that approach. The CWG initially considered two manufacturing strategies for 7.1 % chlorhexidine digluconate: (1) Production and global distribution by a multinational company which offers the potential benefits that the product would likely meet high international manufacturing and quality standards; and the company’s regulatory expertise and distribution network could be leveraged, which could lead to a shorter time to market and wider coverage, and (2) Production and regional distribution by locally owned companies or subsidiaries of multinational companies in LMICs which offers the potential benefits of improved reliability of supply, foreign import savings, development of local capability for innovation, creation of enhanced export capacity, and development of human capital. Local production could also lead to cost savings and improved product quality and regular monitoring of countries’ adherence to quality control standards [8]. Furthermore, local manufacturers would be able to adapt products to local cultural preferences. This approach is consistent with the African Union Pharmaceutical Manufacturing Plan for Africa to strengthen local ability to produce high quality, affordable pharmaceuticals across the list of all essential medicines thereby improving health outcomes and increasing direct and indirect economic benefits in sub-Saharan Africa [9].

The CWG opted for a local production strategy with regional distribution—establishing production bases in selected LMICs and then using them as regional hubs to leverage local manufacturers’ regional distribution networks. Key reasons for the CWG’s choice include a) the product requires neither complex manufacturing processes nor unique production equipment; b) the ingredients, including the active pharmaceutical ingredient, are generic; and c) the primary containers for the product are commonly available in LMICs. Additionally, a local production strategy is consistent with recent movements in LMICs toward strengthening local pharmaceutical industries.

### Linking local production and increased access to 7.1 % chlorhexidine digluconate

Factors that need to be addressed to ensure that local production leads to improved access to high-quality, affordable medicines in LMICs include the capability and capacity of local pharmaceutical manufacturers; quality of local infrastructure (e.g., electrical and water supplies); regulatory and legal provisions; economic incentives and disincentives such as interest rates of loans, duties, and import controls; and market size and competitive landscape [10]. The CWG, therefore, undertook a multi-layered approach to address these factors.

First, the CWG assessed the feasibility of local production by evaluating the aforementioned factors in countries that had expressed interest in implementing 7.1 % chlorhexidine digluconate in their newborn care programs. Based on that assessment, the CWG selected Kenya and Nigeria as initial bases for local production in the sub-Saharan African region. We elicited interest from in-country manufacturers; performed good manufacturing practices assessments of those companies; and identified areas for quality improvement and developed corrective action plans, as necessary. In addition, we conducted market research and assisted the ministries of health and other implementing partners in developing optimal introduction strategies to ensure increased coverage and use of 7.1 % chlorhexidine digluconate. The CWG provides ongoing monitoring of manufacturers to ascertain whether corrective action plans have been properly implemented, and technical assistance until they register their products with their national regulatory authorities. It is anticipated that, over time, national regulatory authorities will take on this role to ensure a high quality product consistent with good manufacturing practices.

One lesson learned during this process was that a government’s desire for local production of medicines is sometimes based on political reasons, even when the country clearly lacks capacity (e.g., a pharmaceutical company, good manufacturing practices compliance), adequate regulatory systems to provide market authorization of the finished product, or sufficient market size for the product to justify its local production. In such cases, the CWG engages in educational dialogue with key government stakeholders. The results of the aforementioned feasibility assessments are quite helpful for those discussions.

Another lesson learned was how to incentivize manufacturers to produce 7.1 % chlorhexidine digluconate. The CWG included them as full members in the group. This enabled manufacturers to gain name recognition, access to key programmatic and policy stakeholders, and potential buyers, and market intelligence which in turn helped them to reduce cost for market entry. In addition, manufacturers that we reached typically had a low capacity utilization rate, and filling ample, unused capacity with product demand which the CWG helped generate worked as a strong incentive. Finally, manufacturers are increasing their recognition of corporate social responsibility. Producing 7.1 % chlorhexidine digluconate to reduce neonatal mortality appealed to their sense of social responsibility.

## Conclusion

Since 2013, the CWG is implementing a local production strategy with regional distribution in some LMICs in order to increase access to 7.1 % chlorhexidine digluconate. Although local production has benefits for public health and economic development of LMICs, several factors—such as capability and capacity of pharmaceutical manufacturers, regulatory and legal provisions, and market factors—must be carefully assessed and addressed to ensure that local production is the correct strategy and that it contributes to improved access to the medicine. To date, this effort has resulted in successful registration of 7.1 % chlorhexidine digluconate by manufacturers in Bangladesh, Kenya, Nepal, and Nigeria. Additionally, the product is now available in domestic and export markets. Data are currently being collected to explore the effect this has had on availability and neonatal mortality rates.

## Abbreviations

CWG: Chlorhexidine Working Group
LMICs: Low- and middle-income countries
WHO: World Health Organization
